# Editorial: New Approaches in Chordate and Vertebrate Evolution and Development

**DOI:** 10.3389/fcell.2022.917101

**Published:** 2022-05-12

**Authors:** Juan Pascual-Anaya, Salvatore D’Aniello, Stephanie Bertrand

**Affiliations:** ^1^ Department of Animal Biology, Faculty of Science, University of Málaga, Málaga, Spain; ^2^ Evolutionary Morphology Laboratory, RIKEN Cluster for Pioneering Research, Kobe, Japan; ^3^ Department of Biology and Evolution of Marine Organisms, Stazione Zoologica Anton Dohrn, Naples, Italy; ^4^ Sorbonne Université CNRS, Biologie Intégrative des Organismes Marins (BIOM), Observatoire Océanologique, Banyuls-sur-Mer, France

**Keywords:** chordate evolution, evo-devo, genomics, vertebrates, cephalochordates, urochordates (tunicates), transcriptomics, phenotypic evolution

**FIGURE 1:**
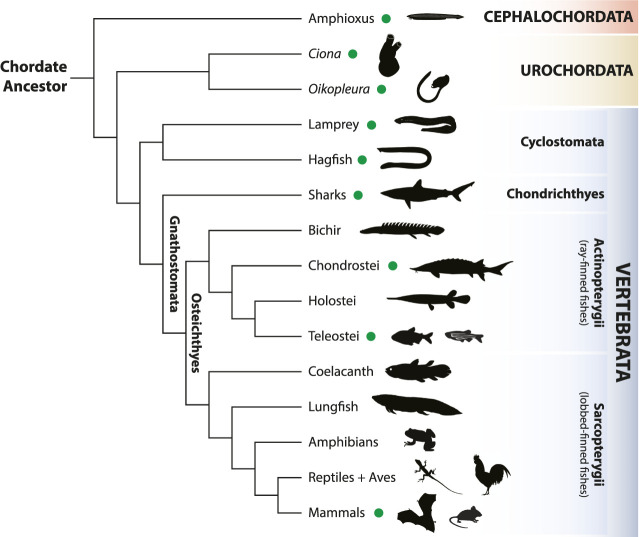
Simplified phylogenetic tree of chordates (vertebrates, urochordates and cephalochordates). Models used in this Research Topic are marked with a green dot. Animal icons are from http://phylopic.org/ under a Creative Commons license.

Consisting of three lineages—cephalochordates (amphioxus), urochordates (tunicates) and vertebrates (including us, humans) (Figure 1), the monophyletic group of chordate animals is defined by the presence, at some stage of their life cycle, of a set of unique and conserved morphological features: a dorsal hollow nerve cord with a notochord just ventral to it, pharyngeal slits and a post-anal tail. Moreover, the last common ancestor of chordates likely possessed a segmented muscular system along the main body axis (Satoh, 2016). Despite these synapomorphies, chordates have largely diversified since their origin around 600 million years ago, especially vertebrates, one of the most successful animal groups on our planet. The evolution of a myriad of morphological novelties (e.g., paired limbs, jaws) are behind chordate and vertebrate diversification since they permitted the adaptation of new species to a vast range of ecological niches.

Two processes are particularly relevant to chordate evolutionary success: the formation of the chordate body plan, and the origin of vertebrate traits. Traditionally, developmental biology and genetics have relied on the use of a handful of model animals (mostly jawed vertebrates like mouse, chicken, frog and fish and few invertebrate animals such as the ascidians *Ciona* and *Halocynthia*), offering a very narrow taxonomic range to tackle these two points. Although evolutionary developmental (evo-devo) studies soon attempted to increase the taxon sampling, these were impaired by the lack of accessible functional genomics techniques readily available for emerging model animals. Consequently, most of the early molecular genetics’ studies in the field of chordate evo-devo consisted of the analysis of a single or a limited number of genes in few species. Therefore, and despite their importance, the mechanisms underlying the origin and evolution of both ancestral traits and morphological novelties of chordates have remained largely elusive. The recent surge and exponential growth of genome assemblies available from a wider set of animal models, together with the availability of the most recent technological innovations in genome editing and whole genome scale high throughput expression and regulation analyses are starting to provide answers to some of the long-standing chordate evo-devo questions.

With this in mind, we present this Frontiers Research Topic, entitled “*New Approaches in Chordate and Vertebrate Evolution and Development*,” consisting of 23 manuscripts, including 14 original research articles and 5 reviews together with reports in several other formats. This fantastic Research Topic introduces multidisciplinary and integrative approaches that, by using the most recent state-of-the-art technologies in genomics, molecular biology, imaging and bioinformatics are contributing towards a better understanding of the mechanistic basis of chordate phenotypic diversity.

Comparative genomics approaches are instrumental to decipher the genomic changes underpinning phenotypic evolution. While vertebrates have traditionally occupied the top position in terms of number and quality of genome sequencing projects, the recent burst of high-quality genome assemblies of less-studied chordate models have provided new opportunities to study genomic evolution at an unprecedented resolution. Aase-Remedios and Ferrier provide a thorough and updated view on how these new genomics and transcriptomics resources, especially in the vertebrate lineage, but also in key invertebrate chordates, have helped us to resolve long-standing questions, such as the exact phylogenetic timing of whole genome duplication events that took place during early vertebrate evolution, or the implications that gene duplicates have had in the evolution of developmental processes. In another review, Herrera-Úbeda and Garcia-Fernàndez address the fundamental question of what is the origin of new genes. They provide an interesting survey on how other processes beyond gene and genome duplication and divergence, such as horizontal gene transfer and the domestication of viruses and transposons, can supply substrates for the evolution of new genes and regulatory sequences, and how the integration of these into existing gene regulatory networks, when transcription factors or transcription factor binding sites are involved, are behind the origin of key vertebrate morphological novelties such as the placenta or the adaptive immune system. Leong et al. take advantage of transcriptomics resources available from different species of chordates, including its sister group, the echinoderms, to measure the degree of derivedness of developmental traits in evolutionary time. To address this question, they conceive a new method called “derivedness index”, which is less constrained than other methods (which use exclusively conserved genomic traits) by taking into consideration lost or duplicated genes (and therefore non conserved), thus permitting to measure the degree of phenotypic evolution through more comprehensive comparative transcriptomics analyses. Tena and Santos-Pereira provide a review on how new technological advances on chromatin conformation studies have enabled us to better understand how the three-dimensional nature of genomes constrain the regulatory landscape of genes, and how changes in these 3D domains can lead to pathological situations, but at the same time provides opportunities for evolutionary innovation.

Cephalochordates, or amphioxus, share with tunicates and vertebrates the oldest common ancestor of chordates. This key phylogenetic position in the chordate tree (Figure 1), together with its slow evolving nature, both at the morphological and genomic levels, turn amphioxus into a unique and crucial animal for understanding the origin of chordate traits. Two original articles now provide new unvaluable resources for comparative developmental studies using amphioxus embryos. First, Carvalho et al., based on confocal imaging of embryological stages of the European amphioxus *Branchiostoma lanceolatum*, used as reference, propose a universal staging system for lancelet species, which they corroborate with comparisons with other amphioxus species. They also propose an unambiguous nomenclature of developmental stages, which will undoubtedly standardize the use of amphioxus embryos from different species across studies. A further extension of this universal staging system for lancelet development is put forward by Bertrand et al., who introduce the first ontology resource for an amphioxus species, termed AMPH, with ontology terms classified as developmental and anatomical specific entities, and hosted in open, public repositories. Importantly, these two amphioxus studies will further facilitate the use of amphioxus in large comparative analysis in evo-devo studies. Lacalli reviews very recent gene expression data on the most anterior region of the central nervous system of amphioxus and provides a thought-provoking article describing the role of heterochronic shifts in the origin of the telencephalon in the vertebrate brain, suggesting that the olfactory function is the driver of such shift. Somorjai et al. show, through careful pharmacological inhibition experiments, the roles that JNK (c-Jun N-terminal kinase) exerts during amphioxus development. Specifically, they find that JNK is particularly important in gastrulation, notochord formation and antero-posterior elongation of the embryo. Also on this Research Topic, Satoh et al. provide the first single-cell RNA-seq analysis in a cephalochordate, the Japanese lancelet *Branchiostoma japonicum*. They generated this preliminary resource from six different amphioxus stages and used it to better understand the expression divergence of the two Brachyury genes present in the amphioxus genome, *Bra1* and *Bra2*. Two last articles focusing on amphioxus provide new methodologies that promise to significantly advance our knowledge of cephalochordates and their use in chordate evo-devo studies. First, Machacova et al. describe a method that combines tissue clearing and light sheet microscopy, which significantly improves the rapid observation of whole-mount post-metamorphic amphioxus, from 1 to 6 month-old stages. Last, Zou et al. provide a simple method for the early detection of mutant and transgenic founders of amphioxus carrying either desired mutations–introduced by genome editing techniques such as the CRISPR/Cas9 technology—or transgenes–introduced by the Tol2 transposase system—, respectively, demonstrating their suitability to be used in amphioxus. This method promises to significantly increase the use of these state-of-the-art technologies for functional genomics studies in amphioxus, enabling a better understanding of the mechanistic processes of chordate development.

Urochordates, or tunicates, are the sister group of vertebrates (Figure 1) and, as such, can provide unvaluable insights into vertebrate origins. Our Research Topic contains two outstanding studies focusing on the fast-evolving appendicularian *Oikopleura dioica*, which represents a unique case to study evolutionary divergence in chordates due to its reduced genome size and large amount of gene losses. The first of these studies, from Calatayud et al., investigates metallothionein proteins, a family of modular proteins with a variable number of cysteine-rich domains. The different number of domains in different genes entail these proteins different binding capacities to transition metal ions. The authors also found that variants of these proteins with different numbers of repetitive domains exist in distinct populations of *O. dioica*. Their work offers a new model in which to study protein modular evolution. Then, Martí-Solans et al. highlight the importance of gene losses and their implications in evolution. These last authors analyzed the Wnt (wingless) gene family, their numbers and expression patterns, finding that few Wnt genes have been retained in *O. dioica*, which therefore contains the shortest, yet functional, Wnt repertoire described so far in chordates. Other three studies focus on the more traditional tunicate model, the ascidian *Ciona* spp.: Marotta et al. identified the gene members of the Activator Protein-1 transcription factor family (AP-1) and their expression patterns in *Ciona robusta*. Given that only single copies of these genes exist in *Ciona*, this enables *Ciona* as a helpful experimental organism to study the evolution of the AP-1 transcriptional complex in chordates; Oonuma et al. provide evidence of a common regulatory network involving *hedgehog* and *FoxA* genes in the origin of midline structures in tunicates and vertebrates, namely the floor plate of the neural tube, plus the hypochord in vertebrates and the endodermal strand in tunicates; last, Olivo et al. review the molecular bases of the pigment cells and the otolith of *C. robusta* in order to understand the evolution of sensory organs in chordates.

Vertebrates represent the most diverse subphylum of chordates (Figure 1), both in terms of morphological divergence as well as genome complexity. The vertebrate lineage is also characterized by key innovations such as the neural crest cells, a craniate head, and the jaw and paired appendages in the gnathostome lineage (Shimeld and Holland, 2000; Holland et al., 2015). Several articles in this Research Topic provide new insights into the evolution of vertebrates, focusing on all major vertebrate branches. In the cyclostome lineage (extant agnathans, or jawless vertebrates), Sugahara et al. report a comparative analysis of gene expression patterns in the dorsal side of the rhombomere 1 in both the lamprey and in the less accessible hagfish. They provide an updated evolutionary scenario for the origin of the cerebellum, involving the presence of a *Ptf1a*-*Atoh1* axis for the specification of inhibitory and excitatory neurons. Kusakabe et al. provide a review of the most recent data in lampreys and sharks concerning the evolution of a type of skeletal muscle, the hypaxial muscles, which are involved in the origin of key novel structures in vertebrates such as the fins and limbs. They propose a new evolutionary hypothesis in which the divergent expression of *Lbx* paralogs is behind the gradual elaboration of skeletal musculature during evolution. Then, Mayeur et al. present an innovative 3D atlas of expression data in the embryonic head of the catshark *Scyliorhinus canicula*. This important adaptation of RNA tomography in a non-model system will bring useful information to other researchers that aim to apply this technique to different chordate species. Continuing with the adaptation of state-of-the-art technologies to emerging experimental organisms, Stundl et al. describe a method for the use of the CRISPR/Cas9 system into a non-model, slow-growing, non-teleost fish: the sterlet sturgeon *Acipenser ruthenus*. Sturgeons belong to the chondrosteans, an important lineage in evolutionary studies of fishes due to its key phylogenetic position as the sister group to two major lineages of ray-finned fishes: holosteans and teleosts. In a proof-of-concept assay, the authors applied the method to successfully mutate the *Tyrosinase* and *Sonic hedgehog* genes. In another research article, Torres-Paz and Rétaux present a powerful tool for experimental embryology, describing a method to grow blastoderm explants (pescoids) and to produce chimeric embryos of the Mexican tetra, *Astyanax mexicanus*, an important evo-devo experimental organism to understand phenotypic evolution. Using these methods, they studied the impact of embryonic and extra-embryonic tissues on cell fate decisions and early developmental processes. To finish, this Research Topic also includes two original articles on the study of bat evolution: first, Ito et al. use advanced computed tomography, called diceCT (from diffusible iodine-based contrast-enhanced computed tomography), to study in detail the 3D prenatal development of various bat species, providing new insights into the evolution of nasal turbinals in bats; and lastly, López-Aguirre et al. studied the development of the humerus in bats, a very specialized bone, using a geometric morphometrics approach to find the presence of fluctuating asymmetry, both on the longitudinal and cross-sectional patterns of humeri, which might be under developmental control of independent mechanisms. They discuss the implications their results might have to understand the selective pressures behind this structure, which is crucial for the specific ecological adaptations of bats.

In conclusion, the collection of articles in this Research Topic introduces the most-updated current state of evo-devo research in chordates, presenting original studies in a wide taxonomic range of animals, and also providing novel methodologies that we hope will significantly improve studies in other evo-devo experimental systems.
